# Flavonoid Profile of the Cotton Plant, *Gossypium hirsutum*: A Review

**DOI:** 10.3390/plants6040043

**Published:** 2017-09-25

**Authors:** Aaron Nix, Cate Paull, Michelle Colgrave

**Affiliations:** CSIRO Agriculture and Food, GPO Box 2583, Brisbane, QLD 4001, Australia; aaron.nix@hotmail.com (A.N.); michelle.colgrave@csiro.au (M.C.)

**Keywords:** biomarker, pigeonpea, *Cajanus cajan*, sorghum, flavone, flavonols, resistance, lepidoptera, host plant interaction

## Abstract

Cotton, *Gossypium hirsutum* L., is a plant fibre of significant economic importance, with seeds providing an additional source of protein in human and animal nutrition. Flavonoids play a vital role in maintaining plant health and function and much research has investigated the role of flavonoids in plant defence and plant vigour and the influence these have on cotton production. As part of ongoing research into host plant/invertebrate pest interactions, we investigated the flavonoid profile of cotton reported in published, peer-reviewed literature. Here we report 52 flavonoids representing seven classes and their reported distribution within the cotton plant. We briefly discuss the historical research of flavonoids in cotton production and propose research areas that warrant further investigation.

## 1. Introduction

Cotton, *Gossypium hirsutum* L., (Malvaceae) is a natural plant fibre of great economic importance grown in more than 50 countries [1,2]. Eighty-seven percent of the world’s cotton growing area occurs in developing countries. In addition to the fibre, cotton seeds offer a supplemental income and a source of protein for human and animal nutrition [1,3]. Almost 97% of world’s cotton production is from upland cotton, *G. hirsutum*, with the remaining production made up by, *G. arboreum*, *G. herbaceum*, and *G. barbadense* [1]. Since the 1950s, the cotton growing area has remained relatively constant between 30 and 36 million hectares. However, global cotton production has increased by 400% from 6.67 million metric tons in 1950/1951 to 26.84 million metric tons in 2012/2013 [1,4].

Flavonoids are one the most important groups of secondary metabolites produced by plants. Once considered waste products stored in vacuoles, flavonoids play important roles in various organs, helping to maintain plant health and function [5]. Flavonoids primarily function as phytoalexins and photoprotectors and modulate the transport of the phytohormone auxin, influencing plant structure [6]. They exhibit a range of biological activities including antioxidant and antifungal properties [7]. Additionally, flavonoids have been demonstrated to affect feeding behaviour of invertebrate pest species [8]. Flavonoid profiles have also been used in chemotaxonomy to identify phylogenetic relationships between plant species [9,10,11,12,13,14].

Early investigations of flavonoids in *Gossypium hirsutum* focussed on the flavonoids from flowers. These studies aimed to identify those compounds responsible for floral colouration [15], determine phylogenetic relationships within *Gossypium* [11,12,13], and investigate the compounds responsible for suppressing larval growth of the tobacco budworm *Heliothis virescens* [16]. Improvements and innovations to maximise cotton production have included research investigating the role of flavonoids in plant defence and fibre production [16,17]. More recently, the focus has shifted to the flavonoids responsible for specific biological activities, the flavonoids involved in leaf reddening, and the role of flavonoids in plant stress response [18]. These have included compounds present in dried flowers (known as *Flos gossypii*) used in traditional Chinese medicine (TCM) [19] and the flavonoids involved in fibre development and colour [17,20].

The purpose of this review is to synthesise published reports on the occurrence and distribution of flavonoids in *G. hirsutum* and to discuss the historical research of flavonoids in cotton and possible avenues for future work. A previous review on the flavonoids from plants of the family Malvaceae identified 23 flavonoids from *Gossypium hirsutum* [21]. Increased interest in the biological activities of flavonoids coupled with improvements in separation and identification techniques over the last 20 years has increased the number of known flavonoids from the cotton plant. Here, we present 52 flavonoids found in cotton representing seven different classes.

## 2. Review General Flavonoid Structure and Biosynthesis

**Figure 1:**
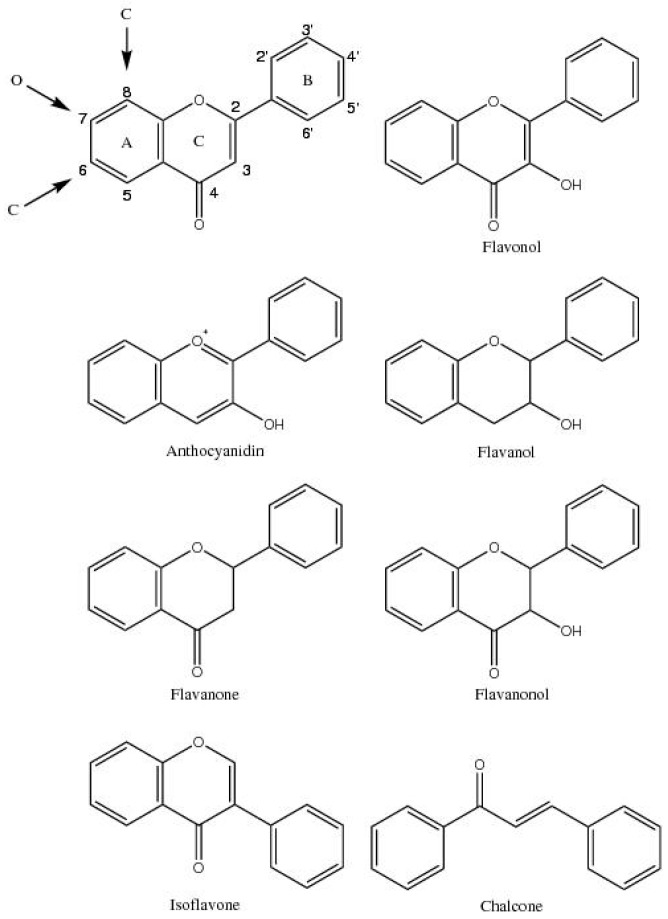
The generalised structure and numbering of flavonoid compounds based on the flavone skeleton (top left) and the generalised structure of various classes of flavonoids.

The general flavonoid structure is a 2-phenylbenzopyranone where the three-carbon bridge between the phenyl groups is commonly cyclised with oxygen [22] (Figure 1). It is the degree of unsaturation and oxidation of the three-carbon segment that distinguishes the major classes of flavonoids based on a C_6_-C_3_-C_6_ flavone skeleton (Figure 1).

There are several classes of flavonoids consisting of flavones, isoflavones, flavonols, anthocyanidins, flavanones, flavanols, chalcones, and aurones, with derivatives found in each class. Glycosidic conjugates are found in relatively high concentrations in plant tissue [23]. Conjugation allows flavonoids to be stored in the cell vacuole while preventing cytoplasmic damage by increasing polarity, rendering the flavonoid less reactive and more water soluble [22].

Flavonoid biosynthesis occurs in the endoplasmic reticulum of vascular and non-vascular plants following the phenylpropanoid metabolic pathway [24,25]. Chalcones important in flavonoid biosynthesis are formed from 4-coumaroyl-CoA and malonyl-CoA in sequential enzymatic reactions with chalcone synthase. Further enzymatic modifications are required throughout the metabolic pathway to produce the other classes of flavonoids with the conjugate ring closure of chalcones resulting in the typical three-ringed flavonoid structure. For a comprehensive description of enzymes and genes involved in the formation of flavonoids in the biosynthetic pathway see [26]. Genetic modification of the flavonoid biosynthetic pathway has been used in various plant species to achieve desirable traits such as increased production of plant-defence flavonoids and to extend floral pigmentation [26].

Manipulating the expression of flavonoids can directly affect plant growth and development, but also indirectly alter auxin transport [27]. Flavonoids, particularly flavonols and isoflavones, are auxin transport inhibitors. These affect plant height, apical dominance, the number of inflorescences, and root development [27,28,29]. The ability to manipulate the expression of flavonoids within plants presents an opportunity to modify natural defence and development, and may provide a novel approach to enhance specific traits from cotton to further increase production.

Investigations on the role of flavonoids in various plant species have focussed on the defensive activity against abiotic stresses and their interactions with other organisms such as herbivores, pathogens, and beneficial bacteria (e.g., *Rhizobium*). Early investigations of *Gossypium hirsutum* were primarily focussed on the flavonoids from flowers, with studies aiming to identify those responsible for pigmentation [15] and to determine the phylogenetic relationship within *Gossypium* [11,12,13]. Subsequent research shifted focus to the biological activities of flavonoids—the role of flavonoids in cotton production and resistance against abiotic and biotic stresses [18,30]. Research in this area aims to identify avenues for increasing fibre yield and reducing losses associated with lepidopteran pests and wilt pathogens.

### 2.1. Flavonoids: Defensive Role in Cotton

Cotton utilises flavonoids as part of a defence mechanism against herbivores by increasing production of toxic flavonoids, inhibiting the larval growth of lepidopteran pests [16]. Phenolic compounds play an important role in providing cotton plants with defences against herbivorous insects [31]. Flavonoids and condensed tannins (proanthocyanidins) act as inhibitors of the larval growth of the tobacco budworm, *Heliothis virescens* [32,33]. The anthocyanin, cyanidin 3-glucoside (chrysanthemin), acts as a feeding deterrent for *Helicoverpa zea* and inhibited the larval growth of *H. zea* and *H. virescens* by 50% over five days of feeding [34]. The chrysanthemin concentration in cotton has been shown to be negatively correlated with larval weights in tobacco budworm larvae, with an median effective dose (ED50) incorporation at 0.07% of the diet [35]. Toxic to 1-day old larvae, isoquercitrin (quercetin 3-glucoside) incorparated at 0.06% of larval diet also reduced the larval growth of 5-day old tobacco budworm larvae [36]. The larval toxicity of the prevalent flavonoids from *G. arboreum* and *G. hirsutum* were compared. Gossypetin 8-rhamnoside and gossypetin 8-glucoside (gossypin) from *G. arboreum* were found to be more toxic to tobacco budworm larvae than any other flavonoid tested (quercetin and its glycosides), with ED50 values of 0.007% and 0.024%, respectively [37]. It should be noted that although these authors did not directly identify gossypin from *G. hirsutum*, it has been reported previously [12,14,16].

The total phenol content (including the concentration of flavonoids) in cotton increases in response to herbivory by *Spodoptera litura*, the Oriental leafworm moth [2]. HPLC analyses revealed distinct elevation in the flavan-3-ol catechin along with the phenolic acids, gallic and caffeic acids, suggesting their role in plant defence mechanisms [2]. Assays have demonstrated varying levels of anti-feedant activity of these compounds toward *S. litura*, with catechin showing low levels of activity. However, significant increases in the production of detoxifying enzymes were observed in the gut of *S. litura* fed on cotton leaves treated with enhanced levels of catechin. This provides further evidence of its role in plant defence mechanisms [2].

Flavonoids aid in resistance to fungal pathogens. Increased resistance to infection from *Rhizoctonia solani* observed in older seedlings of cotton was due to an increase in polyphenols, namely catechin [38]. The higher concentrations of catechin and gallocatechin in wilt-resistant cultivars of *G. hirsutum* were responsible for resistance against the fungal plant pathogen *Verticillium dahlia* [39]. Furthermore, higher concentrations of catechin and gallocatechin, along with isoquercitrin found in young cotton leaves (one to three nodes from the apex) increased inhibition mycelia growth [40].

### 2.2. Leaf Reddening

**Figure 2:**
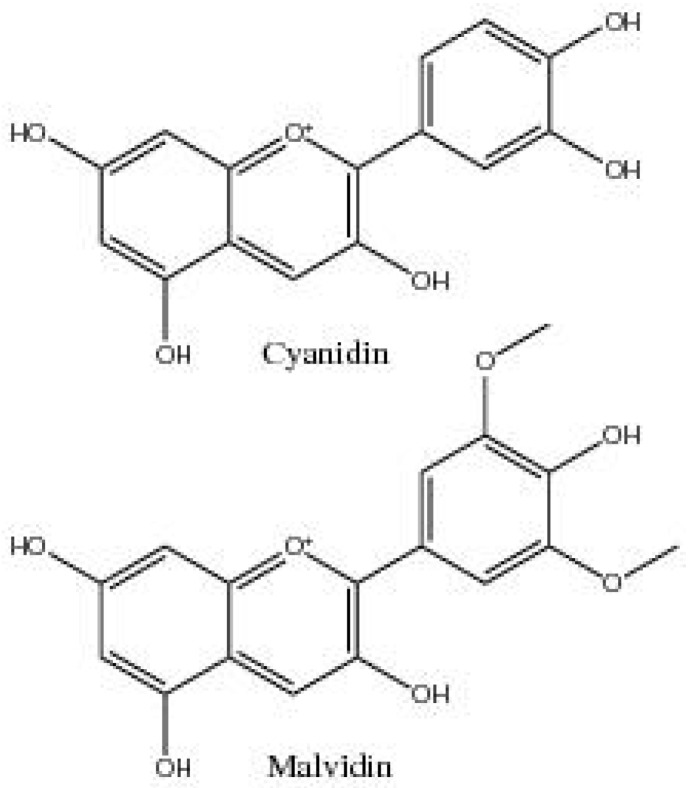
Cyanidin and malvidin, anthocyanidins of cotton.

Leaf reddening and colouration of the leaves in cotton can be due to a physiological response to abiotic stresses, such as Na^+^ accumulation in the soil [41]. Leaf reddening results from a dramatic increase in red pigments and a sharp decline in chlorophyll content, reportedly leading to yield losses of 30–60% [42]. Investigation of the polyphenol complex of cotton leaves showed leaf reddening coincided with an increase in anthocyanin pigments, while other flavonoids and cinnamic acid derivatives were not significantly changed [18]. This increase in anthocyanin pigments was due to a rise in cyanidin glycosides resulting from the transition of malvidin glycoside in green leaves to cyanidin glycosides in leaves exhibiting reddening [18]. This change from malvidin to cyanidin glycosides (O-dihydroxy substitution in the B-ring) (Figure 2) increases the antioxidant and antiradical activity [43], thereby increasing the protective capacity against oxidative stresses [18].

Other research has demonstrated the protective function of cyanidin glycosides in cotton. In response to infection by the bacterial pathogen *Xanthomonas campestris* pv. *malvacearum*, *G. hirsutum* has been shown to produce the sesquiterpenoid phytoalexins 2,7-dihydroxycadalene and lacinilene C [30]. However, these phytoalexins display light-dependent toxicity toward the host plants cells. Cotton produces a dark red flush at the site of infection, and the intensity of this flush is correlated with the level of bacterial blight resistance in isogenic lines [44]. The red pigmentation, caused by anthocyanins at infections sites, plays a protective role in healthy tissues against infection-related reactive oxygen species and light-activated phytoalexins [44]. Further research confirmed the role of epidermal pigments in protecting healthy cells from the plants own light-activated phytoalexins, with red cells exhibiting a 3–4 fold higher absorption of photo-activating wavelengths of light [30]. Analysis revealed the red anthocyanin, cyanidin-3-glucoside (chrysanthemin), and a yellow flavonol, quercetin-3-glucoside (isoquercitrin), as the compounds responsible for the increased absorption capacity [30].

Leaf reddening has also been hypothesised to be a response to insect herbivory signalling high defensive commitment, however, this has not been experimentally verified [45]. Given the role of cyanidin 3-glucoside in defence against *Helicoverpa* species and the role in leaf reddening in response to abiotic stresses, it is not unreasonable to assume that this defensive mechanism against herbivory may exist in cotton.

### 2.3. Role of Flavonoids in Cotton Production

Flavonoids play a role in cotton fibre quality and colour. Brown and green coloured fibres naturally occur in cotton, *G. hirsutum*. Flavonoid accumulation and flavonoid structural gene expression is significantly higher in brown cotton fibre than white cotton fibre, indicating that the flavonoid biosynthetic pathway affects the pigmentation of fibre [20]. Flavonoid metabolism also plays a role in fibre development, particularly during the elongation stage, affecting the quality of fibre produced (both fibre length and micronaire) [17]. The flavanone naringenin is negatively associated with fibre development, with shorter fibres occurring in the presence of elevated levels of naringenin. Mediating the gene responsible for the metabolism of naringenin in order to reduce its levels during fibre development may provide a novel approach for improving cotton fibre development [17].

## 3. Flavonoids of *Gossypium hirsutum* and Their Distribution

Flavonoids are found throughout a variety of plant organs in *Gossypium hirsutum*, from the roots through to the bolls, with the flowers and leaves being richest in diversity (Table 1).

Fifty-two flavonoids have been reported, including the identification of 36 flavonols (Table 2) and five anthocyanidins (Table 3). The remaining known flavonoids include four flavanols (catechins/flavan-3-ols), two flavanones, two flavanonols, two leucoanthocyanidins, and one isoflavone (Table 4). There are no reports of flavones, chalcones, or aurones from *G. hirsutum*.

**Figure 3:**
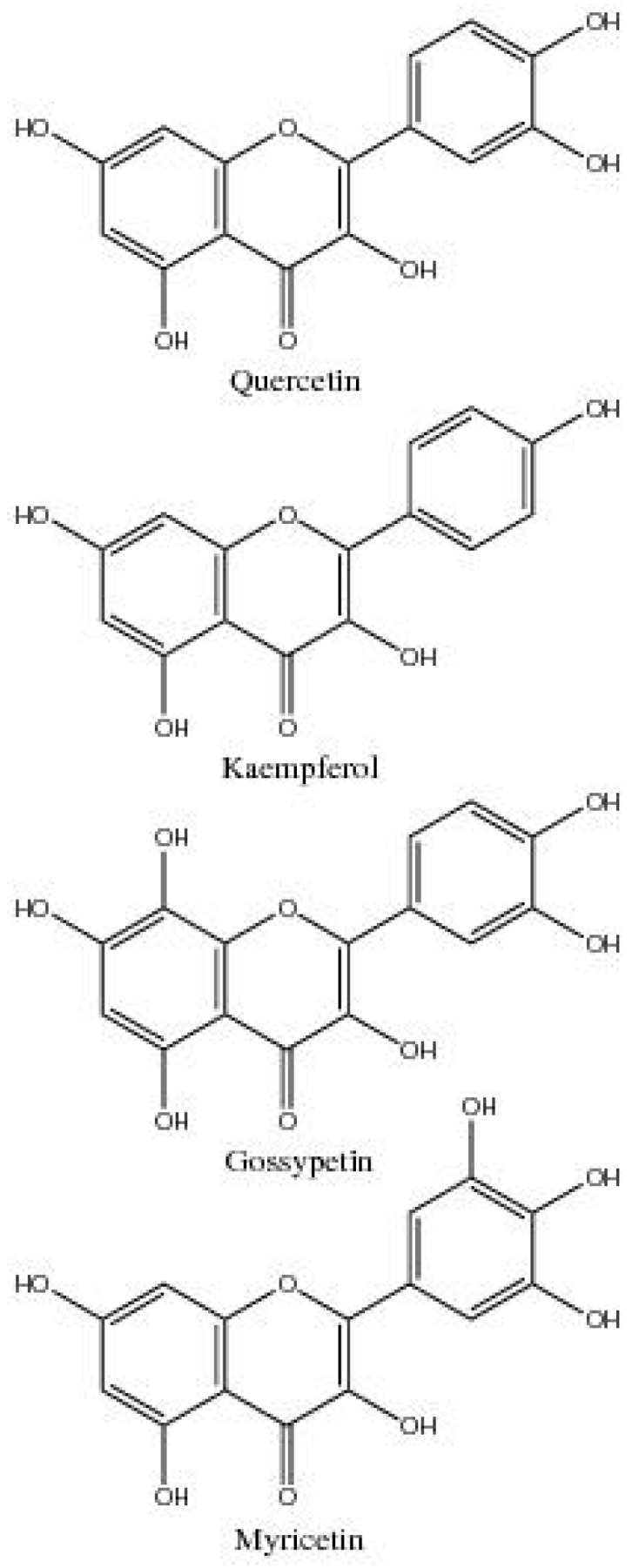
Flavonol aglycones found in cotton.

The flowers of *G. hirsutum* are the plant organ richest in their number of flavonoids, containing 42 of the 52 reported flavonoids. Flavonols are the most represented class of flavonoid within flowers, comprised of quercetin, kaempferol, gossypetin, myricetin, and their conjugates (Figure 3).

The leaves of *G. hirsutum* contain 19 flavonoids spanning five different classes. In contrast, few flavonoids (6–12) are reported from the roots, stem, seed, boll, and fibre of cotton (Table 1).

## 4. Future Research

Although previous research has identified many flavonoids from cotton, there is little information available about their application, or the mechanisms underpinning their biological activity and their interactions, especially in relation to plant = pathogen interactions and pollination events. Here we discuss potential opportunities which support the need for further research regarding flavonoids from cotton, especially in relation to insect pest management. Since the widespread adoption of *Bacillus thuringiensis (Bt)* cotton, there has only been a single report on the role of flavonoids in the plant defence against lepidopteran pest [2] and only a single report on flavonoids from *Bt* cotton [60].

Genetically modified *Bt* cotton has contributed to increased yields and minimised use of pesticides and is an important component of integrated pest management [1]. However, there is a constant risk of pest species developing resistance [61]. For example, *Bt* resistant strains of *Helicoverpa armigera* have been generated under laboratory conditions [62] and genes have been identified from field populations that confer resistance to toxins [63]. Further research to determine the flavonoids that can increase plant pest resistance through moderating feeding behaviour, reducing larval growth, and increasing toxicity toward grazing insects, combined with an ability to manipulate expression levels of these target flavonoids, would provide an additional tool to the cotton industry aiming to stay ahead of rapidly evolving pests.

Flavonoids could be utilised and/or contribute to resistant management strategies in *Bt* cotton/refuge cropping systems by reducing the fitness of lepidopteran pests. Select flavonoids inhibit larval growth, although further research is required to identify those specific to each lepidopteran pest in cotton. Elliger et al. [64] conducted a comprehensive study identifying flavonoids that inhibit growth in the polyphagous crop pest *Helicoverpa zea*. This study examined the effect of 42 flavonoids fed to *H. zea* through an artificially spiked diet, identifying 20 that reduced larval growth 50%. These authors noted a structural commonality between all but one of these inhibitory compounds: each exhibits adjacent (ortho) substitution of phenolic hydroxyl groups. Indeed, seven of these inhibitory compounds are found in cotton (eriodictyol, quercetin, taxifolin, quercitrin, rutin, catechin, and myricetin). Furthermore, several flavonoids previously discussed as exhibiting larval growth inhibition or toxicity toward various lepidopterans (chrysanthemin, isoquercitrin, and gossypin) share this structural trait. Reducing pest fitness through reduced larval growth increases the opportunity for predation or parasitism from beneficial insects, whilst reducing adult size (weight). Again, increasing the fitness (e.g., vitexin) of *Bt* susceptible moths from refuge crops would further improve resistance strategies.

Flavonoids play a role in oviposition for some lepidopterans, either stimulating or deterring, with recognition requiring direct contact with plant material [34]. For example, luteolin 7-malonylglucoside and rutin (and other flavonol glycosides) are stimulants in the black swallowtail, *Papilo polyxenes*, and the monarch butterfly, *Danaus plexipuss*, respectively [34]. *Papilo xuthus* is stimulated to oviposit by the presence of rutin (quercetin 3-rutinoside) on citrus plants, however, it is deterred from ovipositing on the non-host plant *Orixa japonica* by the presence of quercetin 3-(2-β-d-xylopyranosylrutinoside) [65]. Therefore, in this lepidopteran, the switch from oviposition stimulant to deterrent occurs by the simple addition of the sugar xylose to the flavonoid compound [65]. Identifying the flavonoids responsible for deterring and encouraging oviposition in lepidopteran pests in cotton cropping systems could discourage egg lay on cotton while encouraging egg lay on refuge crops.

Selecting cultivars of cotton with elevated levels of defensive flavonoids to incorporate *Bt* genes in, or, manipulating the expression of flavonoids involved in plant defence to higher levels, may provide a novel tool for enhancing the plant defence mechanism. Similarly, determining flavonoids involved in oviposition for lepidopteran pests of cotton could also help in selecting cultivars for *Bt* incorporation that are less attractive to these pests. Furthermore, knowledge on flavonoids that could improve the fitness of *Bt* susceptible moths from refuges, or those which may increase the attractiveness of refuges, could be used to increase susceptible populations, further delaying resistance.

Flavonoids may offer potential as a novel biomarker for identifying natal host crops of cotton invertebrate pests, such as *Helicoverpa* species. Currently in Australia, mandated refuge crops are grown in conjunction with *Bt* cotton. Refuge crops reduce the risk of *Helicoverpa* species developing resistance by increasing the likelihood of resistant moths mating with susceptible individuals [66]. Previous tools employed to identify natal host plants are blunt, relying on the ability to distinguish between C3 and C4 plants groups using stable carbon isotopes. Australian cotton cropping regions grow a range of other crops including the C4 plants maize (*Zea mays*) and sorghum (*Sorghum bicolor*) and the C3 plants cotton, pigeonpea (*Cajanus cajan*), sunflower (*Helianthus annus*), wheat (*Triticum aestivum*), and soybean (*Glycine max*). Pigeonpea is the prefered refuge, as it produces many *Helicoverpa* and therefore requires less area to be grown relative to other refuge crops [66]. Further investigation of the use of stable nitrogen isotope to help distinguish between moths developed on a legume (i.e., pigeonpea) or non-legume (i.e., cotton) C3 plants have been unsuccessful [66]. Combining stable isotope analysis with detection of secondary metabolites such as gossypol has been successful in distinguishing moths developed on cotton with those from non-cotton C3 plants [67,68]. These methods lack the specificity required to identify the natal host crop for these highly mobile pests. It has previously been shown that flavonoid profiles can be used for discriminating phylogenetic relationships and identifying species within genera [9,10,11,12,13,14]. Additionally, invertebrates, including lepidopterans, have been shown to sequester flavonoids from the natal host plant consumed during their development [69,70,71,72,73,74,75]. Despite the potential flavonoids may provide as novel markers with high specificity to host plants, there are no published reports on the application of flavonoids as biomarkers in *Helicoverpa*-cotton cropping systems.

Research into gene expression and the genes responsible for the biosynthesis of flavonoids in cotton were not discussed in detail here. However, they may provide a novel avenue for improving cotton production given the role many flavonoids play in maintaining plant health and function, development, and plant defence mechanisms. Furthermore, research on the sequestration of flavonoids or the flavonoid profiles of cotton and regionally related cropping plants may demonstrate the ability of flavonoids to act as novel biomarkers for natal host plants of cotton lepidopteran pests.

## 5. Conclusions

Here we report a total of 52 flavonoids from cotton, *Gossypium hirsutum*, more than doubling those previously reported [21]. Despite this increase in knowledge on the occurrence of flavonoids within this economic crop, research into the utilisation of this group of endogenous plant compounds is mostly undeveloped. Ongoing research in this area may provide a long-term sustainable technology to support existing approaches for cotton production improvement.

## Figures and Tables

**Table 1:** Distribution of flavonoid classes and number of representatives from plant organs throughout *Gossypium hirsutum*.

Flavonoid Class	Plant Organ						Fibre	Total
	Leaves	Roots/Root Bark	Stem/Stem Bark	Seed	Boll	Flower		
Isoflavones			1					**1**
Flavonols	11	2	1	10	2	29	2	**36**
Flavanones	2	2	1			2	2	**2**
Anthocyanidins	3		2		2	3		**5**
Flavanols	1	4	4	1	4	4		**4**
Flavanonol	2	2				2	2	**2**
Leucoanthocyanidins		2	2		2	2		**2**
**Total**	**19**	**12**	**11**	**11**	**10**	**42**	**6**	**52**

**Table 2:** Distribution of flavonols in *Gossypium hirsutum.*

Name	Structure	Organ Isolated	Reference
**Gossypetin**	3,5,7,8,3′,4′-Hexahydroxyflavone	Flower petals	[14]
**Gossypetin 3′,7-diglucosidoglucoside**		Anthers	[16]
**Gossypetin 3-glucoside**		Anthers	[16]
**Gossypetin glycoside (C7-linked unknown sugar)**		Flower petals	[11]
**Gossypin**	Gossypetin 8-glucoside	Flower petals, anthers	[11,14,16]
**Gossypitrin**	Gossypetin 7-glucoside	Flower petals, anthers	[11,14,16]
**Kaempferol**	3,5,7,4′-Tetrahydroxyflavone	Ovules, Fibre, Roots, Cotyledons, Leaves, Flower buds/petals, Bolls, Seed	[14,17,20,46,47]
**Astragalin**	Kaempferol 3-*O*-β-d-glucopyranoside	Flowers	[14,19]
**Isoastragalin**	Kaempferol 3-α-d-glucofuranoside	Flowers	[48]
**Kaempferide**	4′-*O*-Methylkaempferol	Flower petals	[47]
**Kaempferol C3-linked glycoside (unknown sugar)**		Flower petals	[11]
**Kaempferol 3-diglucoside**		Seed	[49]
**Kaempferol-3-*O*-b-d-(6″-*O*-p-coumaroyl)-glycoside**		Flowers	[19]
**Kaempferol-3-*O*-neohesperidoside**		Seed	[49]
**Nicotiflorin**	Kaempferol 3-rutinoside	Flower petals, leaves, and seed	[11,14,18,46]
**Tiliroside**	Kaempferol 3-*O*-β-d-(6″-*O*-(E)-p-coumaroyl) glucopyranoside	Flowers	[19]
**Trifolin**	Kaempferol-3-*O*-galactoside	Flower petals	[11,13]
**Myricetin**	3,5,7,3′,4′,5′-Hexahydroxyflavone	Ovules and fibres from flower buds and bolls	[20]
**Quercetin**	3,5,7,3’,4’-Pentahydroxyflavone	Flowers/petals, leaves, ovules, fibre, roots, and cotyledons	[14,17,18,19,20,47]
**Hirsutrin**	Quercetin 3-*O*-β-d-glucopyranoside	Flower and Leaves	[48]
**Hybridin**	Quercetin 3-*O*-[*O*-β-d-galactofuranosyl-(l→3)-*O*-β-d-glucopyranosyl-(l→3)-xylopyranoside]	Leaf	[50]
**Hyperoside**	Quercetin-3-galactoside	Flowers	[19]
**Isoquercitrin**	Quercetin 3-β-d-glucoside	Flowers, leaves, cotyledons, and seed	[11,13,14,16,18,19,30,49,51,52]
**Quercetin 3′-glucoside**		Flowers, anthers	[16,19,53]
**Quercetin 3-diglucoside**		Anthers and seed	[16,46,49]
**Quercetin 7-rhamnoglucoside**		Anthers	[16]
**Quercetin C7-linked glycoside (unknown sugar)**		Flower petals	[11]
**Quercetin-3-*O*-neohesperidoside**		Seed	[49]
**Quercetin-3-*O*-robinoside**		Seed	[49]
**Quercimeritrin**	Quercetin 7-glucoside	Flowers/petals	[11,13,14,15,53]
**Quercitrin**	Quercetin 3-rhamnoside	Leaves	[18]
**Rutin**	Quercetin 3-rutinoside	Flower petals, anthers, leaves, hypocotyls, seed, and calli	[4,11,13,14,16,18,49,51,54]
**Sexangularetin 3-glucoside-7-rhamnoside**		Immature flower buds	[55]
**Spiraeoside**	Quercetin 4′-*O*-glucoside	Flower petals, and seed	[14,55]
**Tamarixetin**	Quercetin 4′-methyl ether	Flower petals	[47]
**Tamarixetin 7-glucoside**	Quercetin-4′-*O*-methyl-7-glucoside	Flower petals	[14]

**Table 3:** Distribution of anthocyanidins and anthocyanins in *Gossypium hirsutum*.

Name	Structure	Organ Isolated from	Reference
**Cyanidin**	3,5,7,3′,4′-Pentahydroxyflavylium	Flower petals and leaves	[11,18,56]
**Chrysanthemin**	Cyanidin 3-glucoside	Flower/buds, leaves, cotyledons, anther tissue culture, boll valves, and stem bark	[15,16,30,57,58]
**Gossypicyanin**	Cyanidin 3-*O*-[*O*-β-d-xylopyranosyl-(1→4)-β-D-glucopyranoside	Anther tissue culture, boll valves, stem bark, flowers	[57]
**Ilicicyanin**	Cyanidin 3-xylosylglucoside		[21]
**Malvidin**	3′,5′-Dimethoxy-3,4′,5,7-tetrahydroxyflavylium	Leaves	[18]

**Table 4:** Other flavonoids reported from *Gossypium hirsutum*.

Class	Name	Structure	Organ Isolated from	Reference
**Isoflavone**				
	Genistein	5,7,4′-trihydroxyisoflavone	Hypocotyls	[3]
**Flavanones**				
	Eriodictyol	(2S)-2-(3,4-Dihydroxyphenyl)-5,7-dihydroxy-4-chromanone	Ovules, fibre, roots, cotyledons, and leaves	[17]
	Naringenin	5,7-dihydroxy-2-(4-hydroxyphenyl)chroman-4-one	Hypocotyls, ovules, fibre, roots, cotyledons & leaves	[3,17,20]
**Flavanols**				
	Catechin	(2R,3S)-2-(3,4-dihydroxyphenyl)-3,4-dihydro-2H-chromene-3,5,7-triol	Leaves, hypocotyls, stem, calli, anther, boll valves, stem bark, root bark, and cotton oil cake	[2,3,39,54,57,59]
	(−)-Epicatechin	(−)-cis-3,3′,4′,5,7-Pentahydroxyflavane	Anther, boll valves, stem bark and root bark	[57]
	(−)-Epigallocatechin	(−)-*cis*-3,3′,4′,5,5′,7-Hexahydroxyflavane	Anther, boll valves, stem bark, root bark	[57]
	Gallocatechin	(2S,3R)-2-(3,4,5-Trihydroxyphenyl)-3,4-dihydro-1(2H)-benzopyran-3,5,7-triol	Hypocotyls/stem steles, anther, boll valves, stem bark, root bark	[39,57]
**Flavanonol**				
	Aromadendrin	(2R,3R)-3,5,7-trihydroxy-2-(4-hydroxyphenyl)-2,3-dihydrochromen-4-one	Ovules, fibre, roots, cotyledons, and leaves	[17]
	Taxifolin	(2R,3R)-2-(3,4-dihydroxyphenyl)-3,5,7-trihydroxy-2, 3-dihydrochromen-4-one	Ovules, fibre, roots, cotyledons, and leaves	[17]
**Leucoanthocyanidins**				
	Leucocyanidin	(2R,3S,4S)-2-(3,4-dihydroxyphenyl)-3,4-dihydro-2H-chromene-3,4,5,7-tetrol	Anther, boll valves, stem bark, root bark	[57]
	Leucodelphinidin	(2R,3S,4S)-2-(3,4,5-trihydroxyphenyl)-3,4-dihydro-2H-chromene-3,4,5,7-tetrol	Anther, boll valves, stem bark, root bark	[57]
